# Prayer sign as a marker of disease severity and treatment response in eosinophilic fasciitis: A case series

**DOI:** 10.1016/j.jdcr.2026.02.017

**Published:** 2026-02-19

**Authors:** Daan Jan Willem Rauwerdink, Rutger Casimir Melchers, Koen D. Quint, Anh Ly Nguyen, Deepak M.W. Balak

**Affiliations:** Department of Dermatology, Leiden University Medical Center, Leiden, The Netherlands

**Keywords:** auto-immune disease, eosinophilic fasciitis, fascia inflammation, fasciitis, scleroderma

## Introduction

Eosinophilic fasciitis (EF) is a rare connective tissue disorder characterized by inflammation and fibrosis of the fascia. It presents with an acute onset of painful erythematous swelling, progressive induration, thickening and tightening of the skin and soft tissues of extremities and trunk.^1^ Clinical features may include skin induration with “cobblestone” appearance, leading to a peau d’orange texture of lesional skin, and the groove sign - a visible longitudinal depression overlying superficial veins caused by fascial thickening in elevated extremities.^2^^,^^3^ Although the hands are often spared in EF, forearm tendon synovitis associated with fasciitis can occur and lead to reduced hand mobility and difficulty pressing the palms and fingers together.^4^ This physical sign is referred to as a positive prayer sign.^5^^,^^6^

Herein, we highlight the prayer sign as a key clinical marker of severe disease manifestation in EF and its utility in assessing treatment response. While severe cases can lead to contractures and functional impairment, early and adequate therapy can reverse this sign, preventing long-term disability and restoring full-range of motion of the hands.

## Methodology

A retrospective, IRB-exempt chart review was conducted at an academic dermatology clinic at Leiden University Medical Center spanning January 2020 to December 2024. We included patients with a confirmed primary EF diagnosis based on previously established diagnostic criteria, requiring either the presence of 2 major criteria or 1 major criterion with at least 2 minor criteria, and the exclusion of systemic sclerosis as a diagnosis.^7^

The major criteria included:

1. Swelling, induration, and thickening of the skin and subcutaneous tissue, which could be symmetrical or asymmetrical, diffuse (involving extremities, trunk, and abdomen), or localized (limited to extremities).
2. Fascial thickening accompanied by lymphocyte and macrophage accumulation, with or without eosinophilic infiltration, as determined by full-thickness wedge biopsy of clinically affected skin.

The minor criteria included:

1. Eosinophilia (>0.5 × 10^9^/L),
2. Hypergammaglobulinemia (>1.5 g/L),
3. Muscle weakness and/or elevated aldolase levels,
4. Presence of the groove sign and/or peau d’orange appearance,
5. Hyperintense fascia on T2-weighted MRI images.

Other inclusion criteria for this case series were a documented performance of a prayer sign test before and during treatment, and the availability of photographs.

## Results

A total of 11 patients with eosinophilic fasciitis (EF) were identified, of whom 3 met the inclusion criteria. All 3 patients were female, with a mean age of 45 years (standard deviation: 8.1 years) at diagnosis.

**Fig 1:**
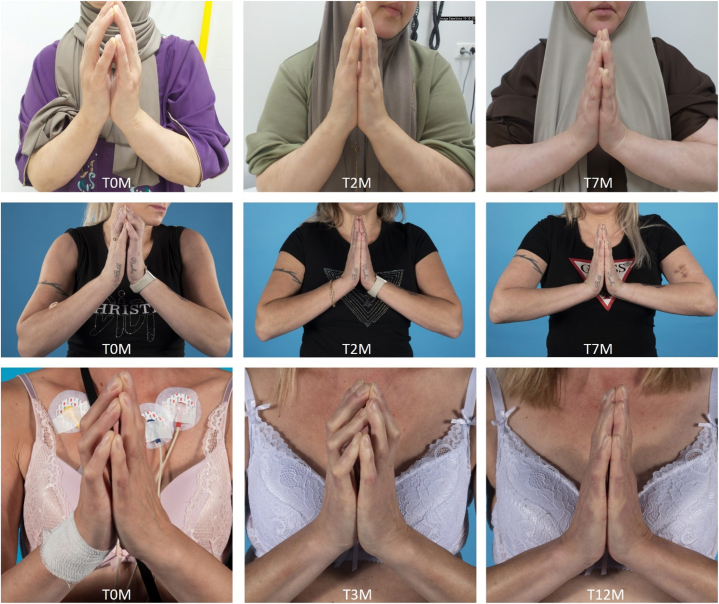
Clinical photographs of 3 treatment-naïve eosinophilic fasciitis (EF) patients demonstrate the inability to press their palms together, a condition referred to as a positive prayer sign. Patient A regained full motion of her fingers and hands after 12 months of treatment. After 2 months of therapy, patients B and C achieved resolution of the prayer sign and regained the ability to fully press their palms together. At 12-month follow-up, patient A regained wrist dorsiflexion up to 115°, while at 7 months, patient B regained full dorsiflexion up to 90°, and patient C up to 100°.

All patients presented with swelling, induration, and thickening of the skin and subcutaneous tissue (Table I). All patients demonstrated a positive prayer sign, characterized by an inability to press the palms together with restricted extension of the fingers. Additionally, they also had restricted dorsiflexion of the wrists (Fig 1). Full-thickness biopsy containing fascia and muscle demonstrated fascial thickening with accumulation of lymphocytes in 1 patient. Among the minor diagnostic criteria, muscle weakness, groove sign and/or a peau d’orange appearance of the skin were present in all patients. Eosinophilia was detected in 1 patient. Additionally, MRI scans were performed in 2 patients, revealing hyperintense fascia on T2-weighted images.

**Table I:** Summary of demographic variables, presence of diagnostic criteria, and type of treatment given for 3 patients with eosinophilic fasciitis

Patient ID	A	B	C
Age at diagnosis (y)	53	44	38
Sex	Female	Female	Female
Major diagnostic criteria for EF			
1. Swelling, induration, and thickening of the skin and subcutaneous tissue, ie, symmetrical or non-symmetrical, diffuse (extremities, trunk and abdomen) or localized (extremities)	Present	Present	Present
2. Fascial thickening with accumulation of lymphocytes and macrophages with or without eosinophilic infiltration (determined by full-thickness wedge biopsy of clinically affected skin)	Present	NA	NA
Minor diagnostic criteria for EF			
1. Eosinophilia >0.5 × 10^9^/L	Present	Absent	Absent
2. Hypergammaglobulinemia >1.5 g/L	Absent	Absent	Present
3. Muscle weakness and/or elevated aldolase levels	Present	Present	Present
4. Groove sign and/or peau d'orange	Present	Present	Present
5. Hyperintense fascia on T2-weighted MRI images	Present	Present	NA
Treatment at diagnosis			
Prednisolone (mg/kg/d)	1	0.2	0.6
Methotrexate (mg/wk)	15	15	15
Time between first symptoms and treatment initiation (mo)	3	4	6

“NA” (not assessed) indicates unavailable data.

*EF*, Eosinophilic fasciitis.

Treatment was initiated within 6 months of symptom onset and consisted of combination therapy with methotrexate (MTX) and prednisolone for all patients. Methotrexate was continued until clinical symptoms had fully resolved. Prednisolone was tapered after 1 month in all patients receiving prednisolone, with a dose reduction of 10 mg per week. After 2 months of therapy, 2 patients (B and C) achieved full resolution of the prayer sign, while patient A regained full motion after 12 months, restoring the ability to press the palms together and fully extend the fingers. At the 12-month follow-up, patient A had regained wrist dorsiflexion up to 115°. At 7-month follow-up, patient B regained full wrist dorsiflexion up to 90°, and patient C up to 100°. Muscle weakness and linear depression (Groove sign) completely resolved in all three patients, and eosinophil levels normalized in patient A, who initially presented with eosinophilia, following treatment.

## Discussion

An infrequent manifestation of EF is forearm tenosynovitis, which leads to difficulty in pressing the palms together and fully extending the fingers. This physical limitation, referred to as the positive prayer sign, reflects underlying fascial thickening and restricted hand mobility. Additionally, a restriction in dorsiflexion of the wrists can be present. Its presence highlights significant functional impairment that can interfere with daily activities.^8^, ^9^, ^10^ The prayer sign therefore serves as an indicator of severe involvement of the upper limbs, corresponding to the limited movement (upper limbs) criterion in the severity classification system proposed by Mazilu et al, where it is scored as 1 point. A total score of ≥2 points is classified as severe. This severity score is useful in clinical practice to assess treatment response and monitor disease progression. Given its association with functional impairment, the prayer sign test may help identify patients at higher risk of further disability.

Although previous studies have noted the occurrence of tenosynovitis in EF and its association with the inability to press the palms together, no studies to date have explored the correlation between resolution of the positive prayer sign and therapy response in EF.^5^^,^^6^ Assessing disease activity and treatment response in EF is challenging, as the abnormalities primarily affect deeper tissues and fascia. Given their depth, these changes may not be readily apparent through visual inspection or palpation of the skin. Also, the lack of reliable objective markers complicate effective monitoring of disease progression and treatment response over time. Within this context, the prayer sign test emerges as a valuable tool for recognizing severe disease involvement, for tracking changes in disease severity as well as identifying early treatment response in patients with EF. Despite its clinical utility, the prayer sign is not diagnostic for EF, as this sign only occurs in approximately 42% of the patients with EF, and the prayer sign can also been seen in other conditions, such as Dupuytren’s contracture and diabetic cheiroarthropathy.^11^^,^^12^ Therefore, its use should be integrated into a comprehensive clinical assessment rather than relied upon as a standalone diagnostic tool.

Although, spontaneous remission of EF has been reported, prompt initiation of therapy remains critical, particularly when a positive prayer sign is present. Active EF can lead to fibrosis, restricted joint movements, and potentially irreversible contractures, resulting in permanent functional impairment.^13^^,^^14^ In our case series, all patients began therapy shortly after symptom onset, reinforcing the importance of early intervention. This case series demonstrates that prompt and adequate treatment can reverse a positive prayer sign and restore hand mobility, followed by a gradual recovery of the wrist dorsiflexion. Notably, 2 patients in our series achieved complete resolution of the prayer sign after just 2 months of therapy, emphasizing its reversibility and value as an early indicator of treatment response. One patient experienced clinical improvement only after a prolonged period, highlighting the heterogeneity in the course of this disease.

In conclusion, the prayer sign test is a valuable, simple and non-invasive clinical marker for identifying severe disease involvement, monitoring disease progression and evaluating therapy response in patients with EF. Further research is warranted to standardize its use, refine its predictive value, and explore its long-term role in the management of EF.

## Conflicts of interest

None disclosed.
